# Standards of Evidence for Conducting and Reporting Economic Evaluations in Prevention Science

**DOI:** 10.1007/s11121-017-0858-1

**Published:** 2018-02-12

**Authors:** D. Max Crowley, Kenneth A. Dodge, W. Steven Barnett, Phaedra Corso, Sarah Duffy, Phillip Graham, Mark Greenberg, Ron Haskins, Laura Hill, Damon E. Jones, Lynn A. Karoly, Margaret R. Kuklinski, Robert Plotnick

**Affiliations:** 1 Edna Bennett Pierce Prevention Research Center, Pennsylvania State University, State College, PA USA; 2 Duke University, Durham, NC USA; 3 Rutgers University, New Brunswick, NJ USA; 4 University of Georgia, Athens, GA USA; 5 National Institute on Drug Abuse, North Bethesda, MD USA; 6 RTI International, Research Triangle Park, NC USA; 7 Brookings Institution, Washington, DC USA; 8 Washington State University, Pullman, WA USA; 9 RAND Corporation, Santa Monica, CA USA; 10 University of Washington, Seattle, WA USA

**Keywords:** Economic evaluation, Cost analysis, Return-on-investment, Research standards

## Abstract

Over a decade ago, the Society for Prevention Research endorsed the first standards of evidence for research in preventive interventions. The growing recognition of the need to use limited resources to make sound investments in prevention led the Board of Directors to charge a new task force to set standards for research in analysis of the economic impact of preventive interventions. This article reports the findings of this group’s deliberations, proposes standards for economic analyses, and identifies opportunities for future prevention science. Through examples, policymakers’ need and use of economic analysis are described. Standards are proposed for framing economic analysis, estimating costs of prevention programs, estimating benefits of prevention programs, implementing summary metrics, handling uncertainty in estimates, and reporting findings. Topics for research in economic analysis are identified. The SPR Board of Directors endorses the “Standards of Evidence for Conducting and Reporting Economic Evaluations in Prevention Science.”

**Electronic supplementary material:**

The online version of this article (10.1007/s11121-017-0858-1) contains supplementary material, which is available to authorized users.

In the nineteenth and early twentieth centuries, many social programs were recognized as worthy endeavors simply because of their beneficent intent. As federal and state governments gradually took over the funding of large-scale social programs, calls for accountability in these public expenditures grew, with an initial focus on documenting the reach of interventions through service delivery (Crowley et al. 2013; Foster et al. 2003; National Academy of Medicine et al. 2016). By the late twentieth century, the focus on accountability expanded from documenting the number of services rendered to also including evidence of impact—preferably assessed through randomized controlled (Baron and Haskins 2011; Fagan and Mihalic 2003; Haskins and Margolis 2015). Even more recently, as evidence of the positive social impact achieved by programs and policies has grown, the focus on accountability has expanded to include evaluations of the economic return from societal investment (Haskins and Margolis 2015; National Academy of Medicine et al. 2016). Increasingly, the public and legislators alike are demanding that public resources be spent on programs and policies where the public benefits outweigh their costs.

This report is concerned with identifying standards for economic evaluations of prevention programs and policies (in contrast with rehabilitation, treatment, or tertiary care). In particular, this work offers standards for the broad field of prevention science—the empirical investigation of preventive strategies and translation of findings to promote well-being. Economic evaluation may be conceptualized as a set of methodological tools for assessing and contextualizing the costs and benefits of an intervention. In this context, economic evaluation is a natural, yet underutilized, methodology of prevention science.

## The Need for Standardization of Economic Evaluations in Prevention Science

Certain methodological, logistical, and ethical issues surrounding these evaluation approaches are unique or especially pertinent when employed to assess preventive interventions (Beatty 2009; Karoly 2011) in part because the economic returns from prevention are often more distal and diffuse than those of downstream interventions (e.g., treatment). From an economic point of view, additional unique issues arise for prevention programs because prevention strategies aim to build lasting human and social capital (e.g., cognitive skills, peer support) that often leads to multiple diverse outcomes. These returns may accrue to systems in both the public and private sectors, across protracted periods of time (Crowley et al. 2013; Foster et al. 2003). Likewise, programs or policies may have long-term consequences, both positive and negative, for different public systems, necessitating broader outcome measurement (Karoly 2011; Lee et al. 2012). Further, most prevention programs are initiated by an agency external to the individual participants (e.g., public schools), and most costs are not borne by the participants themselves (Levin and McEwan 2000; O’Connell et al. 2009). Additionally, many prevention programs affect not only the targeted individual but also those affected by an intervention target’s behavior (e.g., peers, family, authorities, and society at large; Biglan 2016; Coie et al. 1993). Because of the myriad of such considerations and the lack of clear analytic guidelines, economic evaluations of prevention programs can yield varying and inconsistent results—making findings from different studies difficult to compare and of limited value to decisionmakers.

Many federal, state, and local decisionmakers increasingly recognize and seek out high-quality economic evaluations that use consistent methods and reporting standards (Haskins and Baron 2011; National Academy of Medicine et al. 2016). Recognizing this demand, through a multiyear outreach and consensus process, the Society for Prevention Research’s Mapping Advances in Prevention Science (MAPS) III Committee on Economic Analyses of Prevention identified such standards for prevention science. This work was carried out in an effort to bring greater transparency and consistency to the economic evaluation of prevention programs. Although articulating these standards provides a marked opportunity to advance the field of prevention science, it is important to note that economic evaluation is just one of the many considerations for decisionmakers. In particular, policy and practice decisions must also weigh issues of ethics, equity, social justice, and political will in addition to evidence of interventions’ cost, impact, and return on investment (Cookson et al. 2009; Drummond and Jefferson 1996; Zerbe et al. 2010a, b). Below, we provide a brief overview of economic evaluation methods, consider their use in policy-making, and then offer a set of standards to guide their use in the field of prevention science. Importantly, this report does not seek to provide a primer on specific types of economic evaluation. Rather, it provides standards for conducting economic evaluations of prevention programs. Excellent texts on the methods of economic evaluation include Levin and McEwan (2000), Boardman (2011), Drummond (2005), and Zerbe and Dively (1997).

This article begins with a brief overview of methods used in economic analysis, followed by a discussion of how economic analyses are pertinent to policy decision-making. Next, proposed standards are listed and justified, in sections that include standards for framing an economic analysis of prevention, costing a prevention program, estimating economic outcomes for participants, summary metrics, handling uncertainty in estimates, and reporting findings. Finally, next steps are proposed for research in economic analyses of prevention.

## Brief Overview of Economic Evaluation Methods

The resources that society has at its disposal to produce goods and services are limited, but its needs and desires are not. Ultimately, choices must be made about what will be supported and what will be omitted. For many goods, market mechanisms driven by individuals’ and firms’ investment decisions result in the optimal amount and types of goods being produced. However, market mechanisms do not always operate optimally for all kinds of services. For services such as preventive interventions—which can improve health and socioeconomic well-being—market mechanisms often break down. Individual choice in markets struggles to take these “external benefits” into account, resulting in too little investment in prevention. For that reason, governments and philanthropic groups are often the main funders of preventive interventions.

From an economic standpoint, the determination of how best to invest public or philanthropic resources in prevention requires the use of different economic evaluation strategies. These methods include cost analysis, cost-effectiveness analysis (CEA), and benefit-cost analyses (BCA; e.g., budgetary impact analyses, return on investment analyses). Cost analysis is used to determine the cost of implementing a prevention strategy. Cost-effectiveness analysis is a way of comparing the costs of two or more interventions to reduce or produce a single beneficial outcome. Benefit-cost analyses compare the difference in costs of two or more conditions against the differences in benefits of those conditions valued by a common metric (e.g., dollars). Budgetary impact analyses, a form of BCA, are the public-sector analog to private-sector return-on-investment analyses that are used to guide decisions about where to invest resources from the perspective of the government and a business, respectively. Public-sector decisions are often more complex than private-sector decisions because they must consider benefits and costs for *all* members of society, not just those for the entity that funds the program or the individuals who receive it. In this context, benefit-cost analyses may be viewed as a way to calculate society’s “profit” from investing in a prevention program. Although economic evaluations are conceptually simple, the following pages offer information on how they are performed in practice in the context of imperfect information, considering many complex issues and thus stimulating demands to develop standards for producing reliable estimates. Examples of such issues include the following:

- What counts as a cost?
- What counts as a benefit?
- How can the impact of a program’s effects be valued?
- If a program is ongoing, over what time period should costs and benefits be evaluated?
- How can one extrapolate benefits beyond the period in which impact data are gathered?
- How should benefits accruing at different times be valued to reflect the fact that a dollar of benefit received now is worth less than one received in the future?

The answers to these and other questions can support decisions about how best to allocate limited resources for prevention. This report focuses on standards for cost analysis and benefit-cost analysis, although likely informing work in cost-effectiveness analysis indirectly. For those interested in CEA, the Second Panel on Cost-Effectiveness in Health and Medicine has released complementary standards guiding cost-effectiveness analysis (Neumann et al. 2017; Sanders et al. 2016). In the next section, we consider the role evidence from economic evaluations plays in policy and practice decision-making.

## The Use of Economic Evidence in Policy-Making and Program Funding

In an era of evidence-based programming coupled with limited public resources, policymakers and other decisionmakers are increasingly asking for information on economic costs and benefits in order to make funding decisions. Economic evaluations are valued by decisionmakers because they can provide answers to such questions as:

- How much does a program truly cost to different public systems across time?
- What interventions should be used to maximize benefits of interest?
- What is the proper mix of programs to provide to a population?
- What is the optimal way to allocate scarce resources across a population?

The need for evidence-based decisions that take account of both program benefits and costs is greater now than ever. In this section, to recognize the context in which economic evaluations take place and to orient prevention scientists to the value of these standards, we describe: (1) the decisionmakers who are soliciting and who may benefit from economic evaluation, (2) the kinds of economic evaluation information that they request, (3) how economic evaluation contributes to evidence of effectiveness, and (4) examples from the field.

### Which Decisionmakers Want Evidence of Economic Impact?

Decisionmakers interested in economic evaluations of prevention programs include, but are not limited to, elected and politically appointed officials in both the executive and legislative branches of government; program administrators in government; for-profit, not-for-profit, and philanthropic agencies responsible for prevention funding; administrators and evaluators responsible for program implementation; and program developers interested in creating efficient interventions. Economic evaluations are relevant for policy-making and program funding at all levels of government, from city and county-level local jurisdictions, to state branches of government and agency heads, to the executive and legislative branches of the US federal government. Internationally, economic evaluation plays a substantial role in setting funding priorities within nations (e.g., National Health Service in the UK) and for making funding decisions by the World Bank, USAID, the United Nations, and many international nongovernmental organizations (e.g., Bill and Melinda Gates Foundation).

### The Type of Economic Evidence Decisionmakers Want

Sometimes decisionmakers simply want to know the costs of a program. That is, they seek an accurate and comprehensive cost analysis of the total and component resources required to implement a prevention strategy—without an evaluation of the intervention’s impacts. They often compare costs of programs and seek consistent means for doing so. The standards proposed below provide a guideline for producing this kind of information. Sometimes decisionmakers may not know how to request the evidence they want. For instance, they may request evidence of cost-effectiveness when what they ultimately desire is a benefit-cost analysis. Cost-effectiveness analyses are most useful when comparing interventions that affect the same outcome. An example is a study that compares differences in costs between two programs designed to increase graduation rates where the increase in graduation is the only outcome measured. Guidelines for cost-effectiveness analyses are increasingly available (Husereau et al. 2013; Sanders et al. 2016). Decisionmakers often seek instead a more comprehensive benefit-cost analysis that considers the multiple possible benefits of increasing graduation rates, such as improvements in employment and reduced criminal activity that can be expressed as a monetary value. Again, as a result of these needs (and complementary efforts to standardize CEA; Sanders et al. 2016), the following sections focus on standards for cost analyses and benefit-cost analyses as these offer the most comprehensive approach for government decision-making at a macrolevel. In this manner, the standards described here are complementary to efforts that have largely focused on cost-effectiveness or cost-utility analyses and offer tailored standards for the field of prevention science.

### How Economic Evaluations Contribute to Evidence-Based Policy-Making

Scientifically rigorous impact evaluation is a necessary aspect of evidence-based policy-making. There is little objective merit to continuing programs in perpetuity that fail to prevent their targeted social problems. Advocates for evidence-based policy and responsible public spending are increasingly recognizing that programs that are evaluated to assess the rigor of their designs and found not to produce impacts should be reformed or terminated. However, positive impacts at a very high price may not be in the best interests of society.

The use of economic evidence as an important component of policy-making now has bipartisan support (National Academy of Medicine et al. 2016). There is widespread agreement that achieving deficit reduction via across-the-board cuts in funding has not been a successful way to govern (e.g., sequestration; Johnson 2011). Increasing our understanding of programs with no positive impact or those that produce impacts at too high a price would allow decisionmakers to make more efficient decisions to reduce deficits—cutting only programs that fail to yield a positive return on investment. Many pieces of legislation that authorize or appropriate funds for social programs now require high-quality economic evaluations of those programs (Vandlandingham 2015). As a result—and with the federal debt likely to continue shaping policy debates—legislators and other decisionmakers would be well served by increased rigorous economic evaluations of the programs they fund. Augmenting the availability and utility of such evaluations ultimately requires increased standardization. Below, we provide examples of the growth in demand for economic evaluation, which in turn provides the broad context within which the MAPS III Standards were produced.

### Examples from the Field

#### The Commission on Evidence-Based Policymaking

An example of the growing influence of economic evaluation is the new federal Commission on Evidence-Based Policymaking, signed into law in 2016. Introduced by the Republican Chairman of the Ways and Means Committee (Paul Ryan; R-Wis.), and the ranking Democrat on the Senate Budget Committee (Patty Murray; D-Wash.), the Commission is composed of 15 unpaid members, academic researchers, data management experts, and program administrators appointed by the President and Majority and Minority Leaders of the Senate and House of Representatives. The Commission is charged with three tasks:

1. Perform a study of the federal government’s data inventory, data infrastructure, and statistical protocols in order to facilitate program evaluation and policy-relevant research. With this task in mind, the Commission is required to make recommendations on how best to incorporate outcomes measurement, and to institutionalize randomized controlled trials and rigorous impact analysis into program design;
2. Explore how to create a clearinghouse of program and survey data to increase the use of evidence-based policy-making; and
3. Submit a report to the President and Congress detailing the Commission’s findings and recommendations.

The Commission’s responsibility is to “determine the optimal arrangement for which administrative data on Federal programs and tax expenditures and related data series may be integrated and made available to facilitate program evaluation, policy-relevant research, and benefit-cost analyses.” It is not surprising that Commission members want to be sure that Congress knows how economic evaluations can be conducted with existing federal data, because members of Congress want to know the dollar value of benefits produced by federal programs. Based on the number of laws that now require rigorous evaluations and economic evaluations of social programs, combined with the attention that will be given to economic evaluation by the Ryan/Murray Commission, it is likely that the influence of economic evaluations in the federal government will continue to grow.

#### The Office of Information and Regulatory Affairs at the Office of Management and Budget (OMB)

This agency has long provided guidance to federal agencies on including economic evaluation as part of regulatory impact analyses for economically significant rules. The OMB circular explaining the use of economic evaluations in federal rule making provides guidance on how to: (1) quantify and monetize the benefits and costs of regulatory actions; (2) evaluate nonquantified and nonmonetized benefits and costs; and (3) characterize uncertainty in benefits, costs, and net benefits. The purpose of the guidance is to inform agency decisions in advance of regulatory actions and to ensure that regulatory choices are made after appropriate consideration of the likely consequences. To the extent permitted by law, agencies should proceed only on the basis of a reasoned determination that the benefits justify the costs (recognizing that some benefits and costs are difficult to quantify). Economic evaluation within regulatory analysis also has an important democratic function. It promotes accountability and transparency and is a central part of open government. Important goals of economic evaluations included as part of these regulatory analyses are to establish whether federal regulation is necessary and justified to achieve a social goal. Further, they are used to clarify how to design regulations in the most efficient, least burdensome, and most cost-beneficial manner.

#### The Congressional Budget Office (CBO)

One of the major duties of the CBO is to “score” the cost of legislative proposals (e.g., project the impact of proposed legislation on government spending and revenue). Many of the proposals the CBO scores address social policy—either new social programs or reforms of old programs. In these cases, the CBO is sometimes willing to score savings against the cost of the program if there is strong evidence, usually from randomized controlled trials (RCTs), showing that the program results in government savings because some social problem is reduced in magnitude. If the study includes the kind of economic evaluation using standards we propose, and if the evaluation shows government savings, the CBO would be all the more likely to reduce its estimate of program costs to reflect the savings. In early drafts of the Affordable Care Act, for example, the CBO (as well as the OMB) scored savings as a result of an appropriation for spending on the Nurse Family Partnership home visiting program, a program with evidence from three RCTs of impacts that result in government savings in healthcare and other programs. The Coalition for Evidence-Based Policy summarized both the CBO and OMB positions on using evidence from social science research in scoring; their summary includes a CBO letter explaining their position and an outline of the OMB’s position (Coalition for Evidence-Based Policy 2011).

The fact that both CBO and OMB score savings achieved by social programs if the savings are demonstrated by RCTs suggests that, under CBO scoring rules, successful social programs have the potential to reduce the official cost estimate of bills creating, reforming, or expanding social programs. This is an important consideration because under congressional budget rules, legislation must be paid for by either increased taxes or cuts in other programs. If a social program that Congress appropriates $1 billion to support is scored by the CBO as saving $200 million in government spending (e.g., through reduced Medicaid spending), then Congress would need to identify only $800 million rather than $1 billion to offset the program cost. Thus, unlike most other types of spending, prevention programs that save government money have the potential to apply the saving directly to the estimated net costs of the program even during budget allocation.

#### Washington State Institute for Public Policy

At the state level of policy-making, economic evaluation similarly plays an important role in the policy-making process. Created by the Washington State legislature in 1983, the Washington State Institute for Public Policy (WSIPP) is one of the earliest examples of using benefit-cost analysis to advise state policymakers on a systematic and routine basis. WSIPP is a nonpartisan agency governed by a board of directors with representatives from the legislature, the governor’s office, and public universities. The agency has a director and a staff to perform the policy analyses requested by the state legislature. In a typical analysis, the agency is asked by lawmakers to investigate a problem the legislature wants to address and provides them with an assessment of programs that research has shown to effectively attack the issue. The assessment includes an analysis of the costs and benefits produced by the programs the legislature may support. The agency provides state policymakers with a list (referred to as a “portfolio”) of programs and the costs and benefits they produce, which policymakers can use as a tool in fashioning legislation. The benefit-cost information provided to policy makers by WSIPP staff has become a major part of the legislative process in Washington, owing to the reliability of information WSIPP has provided over the years.

#### Results First Initiative

Nongovernmental organizations (NGOs) also play an important role in the advancement of economic evaluation in policy making. Building on the WSIPP program, the Pew-MacArthur Results First Initiative is a large-scale attempt to help states develop a framework to bring evidence, especially benefit-cost studies, to bear on policy choice (Pew Charitable Trusts 2015). Early results in the six states that joined the initiative in 2013 show that these states have directed $38 million in funding to projects that have been shown by rigorous evaluation to be effective (including cutting ineffective services); analyzed a host of criminal justice policies in the search for ways to save money; and passed legislation that makes following the Results First framework in deliberations on policy and budget a legal requirement (Pew Charitable Trusts 2015). Given the scope of the Pew-MacArthur initiative, it is likely that Results First will continue to boost the use of benefit-cost analysis in helping state legislators make program funding decisions.

#### Pay for Success Financing

Turning to the private sector, the Pay for Success movement is an example of how private investors use benefit-cost analyses to fund prevention programs (Crowley 2014; Edmondson et al. 2015). The basic premise of Pay for Success is that private-sector investors will finance the upfront costs of prevention programs that promise a high fiscal benefit (Liebman and Sellman 2013). If that benefit is realized, the government pays the investors a return on their investment in addition to reimbursing them for their costs. In a typical arrangement, an intermediary (such as a research firm) obtains financing from private sources, including wealthy individuals, corporations, or foundations, and then turns those funds over to a government or private agency to pay for program implementation and evaluation. The intermediary usually signs an agreement with the private funder and a government agency, stipulating the expected impacts, how these impacts will be assessed (often by conducting an RCT) and by whom, and how savings will be realized and computed. The agreement asserts that if a program produces the impact specified in the agreement, the government will pay the intermediary, who in turn reimburses the investors. If there are actual government savings above the cost of the program, that amount can be paid to the investors who will then turn a profit on their investment.

Economic evaluation is central to the Pay for Success contract. As part of the agreement, the anticipated costs of the program are described in detail. Similarly, the expected savings, and the specific program budgets that will realize the savings, are described. The bottom line in Pay for Success, as in any benefit-cost analysis, is whether benefits exceed costs (Crowley 2014; Roman 2014). Over 50 programs worldwide now are based on Pay for Success principles, due primarily to two characteristics of the approach. A major impact of deficits in many nations has been constrained funding growth, or even budget cuts, often by more or less haphazard reductions in spending. Even so, social needs continue and can often be addressed effectively by social programs, especially programs backed by rigorous evidence of positive outcomes. Given the current realities of government finance, new sources of operating capital are needed. Thus, bringing new funders into the financing arrangements for social programs meets a growing need.

There is room for skepticism about whether Pay for Success will lead to more effective social programs (Stidd 2013). An important issue is whether government decisionmakers will fully embrace Pay for Success due to its complexity. Another complication is that the benefits of most programs accrue to more than one government budget and at staggered time points. Savings in an early childhood program, for example, could accrue to public schools, child protection agencies, juvenile justice agencies, and health agencies, thereby requiring these agencies to work together to figure out how to attribute the savings (Crowley 2014; Lantz et al. 2016). The field is very early in the process of trying to make Pay for Success programs work effectively, so no final judgment about its ultimate value is in order. Nonetheless, benefit-cost analysis is the platform on which Pay for Success is constructed and high-quality economic evaluation will support the most impactful deals.

#### National Academies of Science, Engineering, and Medicine

The National Academies has supported several efforts to strengthen the role of economic evaluation of interventions for children, youth, and families. After holding workshops in 2009 and 2013 on, respectively, Strengthening Benefit-Cost Analysis for Early Childhood Interventions and Standards for Benefit-Cost Analysis of Preventive Interventions for Children, Youth, and Families, the National Academies convened a consensus panel in 2014 to make recommendations to improve the quality, utility, and use of research, evaluation, and economic evidence when considering investments in children, youth, and families. The panel’s report, *Advancing the Power of Economic Evidence to Inform Investments in Children, Youth, and Families* (National Academy of Medicine et al. 2016), was issued after taking into consideration the perspectives of and actions that can be taken by prevention researchers, economic researchers, implementation researchers, evaluation scientists, implementers, and those engaged in making decisions about policies and investments. It concluded that “the greatest promise for improving the use of economic evidence lies in producing high-quality and high-utility economic evidence that fits well within the context in which decisions will be made.” (National Academy of Medicine et al. 2016, p. 35).

The National Academies report includes recommendations for producing high-quality evidence through the application of best practices for conducting and reporting the results of economic evaluations. This work builds on those recommendations. It also makes a number of recommendations about actions that a diverse set of stakeholders can take to improve the production, utility, and use of economic evaluation evidence. These recommendations include, among others, investing in a coordinated data infrastructure supporting the development of high-quality economic evidence, increasing education and training in economic evaluation methods, and developing multistakeholder partnerships that ensure multiple perspectives are incorporated into action plans related to economic evidence. This important report, which considers a broader set of investments in children, youth, and families than the prevention focus of this report, has helped move the field forward with its dual emphasis on producing high-quality economic evaluation evidence that is attentive to the realities of policy and decision-making in which it is intended to be used. Further, the standards below are consistent with the recommendation by the National Academies to standardize economic evaluation methods to increase the availability of high-quality economic evidence.

## Standards of Evidence for Economic Evaluation of Prevention

The growing importance of economic evaluation in policy and practice motivated the MAPS III Committee’s charge to provide guidance and identify standards for economic evaluation of prevention. Below and listed in Table 1, we propose standards for (1) framing an economic evaluation, (2) estimating intervention costs, (3) valuing effects, (4) producing summary metrics, (5) handling estimate uncertainty, and (6) reporting findings. These standards were developed based on the growing demand for evidence of prevention economic impact, described above, with a focus on increasing the comparability and quality of economic evaluations of prevention programs. These standards build on previous efforts to identify best practices and guidelines in other fields or areas of study. In particular, this work builds on recent efforts by the National Academies, which outlines recommendations for economic analyses of programs for children, youth, and families (National Academies of Medicine 2015). Further, this work complements the guidance by the Second Panel on Cost Effectiveness in Health and Medicine (Sanders et al. 2016), which updated the first panel’s recommendations for general guidance in cost-effectiveness analysis and, in our discussion of reporting, efforts by groups such as the ISPOR Health Economic Evaluation Publication Guidelines Good Reporting Practices Task Force (Husereau et al. 2013). Although these efforts are of benefit to prevention scientists conducting economic evaluations, they do not explicitly seek to standardize economic evaluations of preventive interventions in a manner that will allow for the need comparably and transparency. Thus, it is in the context of the pressing need for a consistent methodology across prevention studies to produce high-quality comparable evidence of economic impact these standards are set. We outline these standards in order to support researchers’ efforts to understand costs, benefits, and return-on-investments of preventive interventions.

**Table 1:** SPR standards for economic evaluation of prevention programs

Section and related standards
I. Standards for framing an economic evaluation
I.1. State the empirical question being addressed by the economic evaluation
I.2. Describe in detail the program being evaluated and its comparator
I.3. Describe the evaluation of the prevention program’s efficacy or effectiveness in terms of its impact on behavioral and other noneconomic outcomes
I.4. Determine and describe the perspectives from which analyses are conducted
I.5. Describe the time period and systems included and excluded in the evaluation
II. Standards for estimating costs of prevention programs
II.1. Plan cost analyses prospectively and then conduct them concurrently with program trials
II.2. Use an ingredients method in cost analysis
II.3. Describe comprehensively the units and resources needed to implement the intervention, disaggregated by time
II.4. Include resources consumed but not paid for directly
II.5. Resources needed to support program adoption, implementation, sustainability, and monitoring should be included in cost estimates
III. Standards for valuing effects of prevention programs
III.1. Estimate findings for each program outcome separately from benefit estimates and describe the context of the evaluation
III.2. Balance the rigor of direct valuation of outcomes with the validity of indirect valuation in contemporary society
III.3. Consider outcomes with negative monetary values as negative benefits rather than part of program costs
IV. Standards for summary metrics
IV.1. Estimate all costs and benefits in current monetary units or in monetary units for the most recent year available
IV.2. Estimate current values for benefits and costs that accrue over time by selecting and reporting a reputable discount rate
IV.3. Estimate and report the total, per-participant average, and marginal costs of the program
IV.4. When applying benefits across multiple outcomes to generate total economic values, avoid double counting of economic impact
IV.5. Use the net present value with a confidence interval as the principle summary metric of benefit-cost analyses
IV.6. Describe the advantages and limitations of any additional summary metrics that are included in the evaluation. Some metrics should be used only when certain conditions are met
V. Standards for handling estimate uncertainty
V.1. Test the uncertainty in estimates and report the manner in which it is handled
VI. Standards for reporting economic evaluations
VI.1. The principle of transparency should guide the reporting of economic evaluation results
VI.2. Use a two-step reporting process that summarizes the most essential features and results of an evaluation in a table or brief report and offers supporting technical detail elsewhere
VI.3. When Monte Carlo analysis is performed, present a histogram of the net present value distribution as well as the percentage of simulations that return a positive net present value

Corresponding description of each standard can be found within the text under the standard number

## Standards for Framing an Economic Evaluation

The first step in conducting any economic evaluation is to define the scope of analysis. This section provides guidance on the major elements to consider when framing the evaluation. Economic evaluation is best thought of as a tool to help decisionmakers allocate scarce societal resources and to assist administrators in selecting interventions that meet their objectives. The manner in which the evaluation’s findings will be used drives many decisions during the evaluation and therefore should be stated clearly. In recent years, those interested in conducting economic evaluations have expanded to include both state and federal government as well as nongovernment researchers, international interests, and politically motivated interest groups (Pew Center for the States 2010; Vandlandingham 2015).

### Standard: State the Empirical Question Being Addressed by the Economic Evaluation

In order to frame an economic evaluation, the empirical question of interest must first be clearly articulated. From this question, many of the other aspects of the evaluation’s scope become clear. Further, the analyses themselves will be heavily influenced by the question of interest. If the question is, “What is the cost of this school-based intervention?,” then there may be no need for a full benefit-cost analysis. In contrast, if the primary question is, “What is the impact of this family-based intervention on costs to the criminal justice system?,” the analysis is likely going to require substantial evaluation of impacts on law enforcement, courts, and detention systems. Asking, “What is the economic benefit of this high school dropout prevention program to the participant?” versus “What the economic benefit of this high school dropout prevention program to society?” will result in different analyses with different findings. Both will include a benefit-cost analysis, but one will focus on the impact of the program on the participant (e.g., increased earnings) while the other will focus on the impact on society (e.g., increased tax revenue). In this context, the question should be clear to avoid unintentionally conducting evaluations that fail to answer the intended question of interest.

### Standard: Describe in Detail the Program Being Evaluated and Its Comparator

An economic evaluation should describe or reference the prevention program in sufficient detail that readers who are not experts in the problem behavior or familiar with the specific program can understand its purpose and essential elements. The description should include at least the following:

- The program’s *primary goal* (e.g., delinquency prevention, ensuring full-term birth). If the program has multiple goals (e.g., improving infant health and reducing unplanned pregnancies), all goals should be operationalized.
- The program’s *theory of change*. Identify the activities involved, stages over which these activities take place, and potential direct and indirect effects of the program as comprehensively as possible.
- The *nature of the program*. Identify eligibility criteria (e.g., universal, selective, or indicated), delivery setting (e.g., school, home), population served, specific services (therapies, education, training), protocols, duration, and other salient characteristics.
- The *time period*(s) in which the program was conducted and a description of the *location*(s) and *context*(s) (e.g., participant, provider, and community characteristics) in which the program was implemented. If multiple sites were involved, describe any major differences in how the program was carried out, who was served, and how it was administered. Provide a rationale for whether the analysis should be conducted separately by site.
- The *comparison condition*, that is, whether the alternative to the focal program is no program, an alternative intervention, etc. Description of this comparator is just as important as the description of the program. A program’s economic evaluation results can change dramatically if the comparison condition changes.

### Standard: Describe the Evaluation of the Prevention Program’s Efficacy or Effectiveness in Terms of Its Impact on Behavioral and Other Outcomes

For economic evaluations beyond cost analyses, decisionmakers need to understand what impact estimates are included in the economic evaluation (e.g., education, criminal justice, health). The full range of theoretically possible impacts as well as which ones were measured and whether they were monetized should be described. The information should address the following issues:

- The *research design* that yielded the effects to be monetized in the economic evaluation (e.g., randomized controlled trial, propensity score analysis, instrumental variables analysis, “natural” experiment, regression discontinuity). See Gottfredson et al. (2015) for a discussion of standards for evidence of prevention program effects.
- *The sample* considered in the evaluation. This information includes the size, demographic characteristics, and risk status. The representativeness of the sample(s) used in the analysis should be clearly described.
- Which *potential impacts* were measured and which were not, and the estimated size of each impact and its level of statistical significance. Discuss whether there is reason to believe that some unmeasured impacts could be substantial, so that their omission might skew the findings. Briefly describe methods and instruments used to measure impact.
- Information about the *data quality* used to measure the program’s effects should be outlined. This information includes whether the data come from surveys, administrative records, observational reports, or some other source(s). The quality of the data should be discussed (e.g., whether surveys were validated; how the quality of administrative data was assured; how complete the data are, and how missing data were handled).
- The *quality of the evaluation study*, including follow-up and attrition rates by condition at each measurement time point should be outlined (e.g., CONSORT diagram).
- *Limitations*, if any, on the ability to draw causal inferences from the analysis.
- *Concerns*, if any, about the internal and external validity of the findings.

### Standard: Determine and Describe the Perspectives from Which Analyses Are Conducted

The analytic perspective within an economic evaluation guides decisions around how cost and benefits are determined. For instance, the resources needed to implement a program may represent a substantial cost to the implementing agency but an inconsequential cost to the participant. The societal perspective, most common in benefit-cost analyses, considers the economic impact on society as a whole (Vining and Weimer 2010). Although the societal perspective has value for understanding comprehensive costs and the total benefits of a program, additional detail about who bears the costs and who receives the benefits is often needed to inform program planning and policy making (National Academy of Medicine 2014). Many stakeholders within society are affected by prevention programs on both the costs and benefits sides (O’Connell et al. 2009). To determine where a prevention program may be housed, who would be willing to fund the program, and what groups will receive its benefits, disaggregated information about costs and benefits is often necessary and valuable to decisionmakers (Levin and McEwan 2001; Welsh 2014). A common approach is to compute benefits and costs separately for program participants and taxpayers, as well as for society as a whole.

### Standard: Describe the Time Period and Systems Included and Excluded in the Evaluation

Although comprehensive analysis of an intervention’s impact on all public systems and the entire life course of participants would be ideal for a societal economic evaluation, most evaluations are limited by design or feasibility. For example, Pay for Success contracts have a specific time period for repayment to the investor that limits the period that can be considered in evaluation of the economic worth of a possible preventive intervention. When an evaluation of a program is undertaken from a government perspective, the benefits should be specific to the government sector. When outcomes from a prevention program have consequences for government revenues, expenditures, or transfers, the economic values that are attached should capture only the fiscal component for the relevant government jurisdiction (e.g., federal, state, or local level) or agency. In addition, when prevention programs have impacts that affect tax revenue (e.g., increased employment), these economic benefits should account for the administrative costs (e.g., cost of monitoring and compliance). Sometimes, it is simply not feasible for an economic evaluation to consider distant-future time periods or particular public systems. In these cases, the evaluation should state explicitly which time periods and sectors are covered and acknowledge possibly relevant periods and sectors that are not addressed.

## Standards for Estimating Costs of Prevention Programs

The evaluation of program costs and resource needs is essential as a basis for benefit-cost analysis, but also because detailed analysis of costs can improve subsequent program planning and implementation. A comprehensive analysis of the cost of implementing a prevention program is important to decisionmakers’ efforts to determine whether to adopt a program. This information has utility even in the absence of a benefit-cost analysis. However, program costs are often calculated or estimated retrospectively, if at all. Additionally, program costs are often reported as a sum of direct expenditures and may neglect resources such as volunteer time, participant time, or program donations. Evaluations sometimes neglect an accounting of the infrastructure costs to adopt programs, integrate them with existing organizational activities, and sustain them over time. Finally, the methods used to calculate costs are highly variable, which reduces our ability to compare costs across programs or to generalize program costs to new settings.

The process of understanding a program’s resource needs continues to be one of the most consistently neglected yet addressable areas of program evaluation (Crowley et al. 2012; Foster et al. 2007; National Academy of Medicine 2014). Cost analyses specify the resources required to replicate a program’s effectiveness (Gold et al. 1996). High-quality cost analyses move past simple accounting exercises and link resource consumption to specific program activities, infrastructure, and capacity-building operations (Frick 2009; Levin and McEwan 2000). Cost analyses also provide the foundation for economic evaluations linking intervention resources to impacts (i.e., cost-effectiveness, benefit-cost analyses). The information they provide about the total, per-participant average, and marginal cost of carrying out an intervention can be particularly informative to program planning efforts (Anderson et al. 1998; Yates 1996). Including information about how resources are used in various intervention activities, when they are needed, and who bears the cost further increases the utility of information gained in a cost analysis. The standards described in this section address elements essential to high-quality cost analysis.

### Standard: Plan Cost Analyses Prospectively and then Conduct Them Concurrently with Program Trials

Cost analyses require considerable planning for effective execution (Beatty 2009). Retrospective analyses generally fail to capture all program costs due to gaps in the measurement of resources needed to implement the intervention and their prices (Crowley et al. 2013; Foster et al. 2007). Prospective planning before a study starts allows evaluators to identify essential program activities, such as curriculum delivery or home visits, that consume resources. Further, this planning provides the opportunity to ensure that activities supporting program infrastructure or capacity-building are properly included in the analysis (e.g., management or fidelity monitoring systems). This early identification is essential to capturing program costs accurately (Crowley et al. 2012; Yates 1994). Once identified, measurement protocols can quantify actual labor, equipment, and space requirements in real time and allocate them to the major operations essential to high-quality program implementation and impact, consistent with the program’s theory of change (French et al. 1997; Frick 2009). Such cost analyses are crucial to replicating program impact because they help ensure that adequate resources are provided (Belfield and Levin 2013).

### Standard: Use an Ingredients Method in Cost Analysis

When conducting a cost analysis, researchers should consider each resource required to replicate an intervention with fidelity (Drummond et al. 1993; Gold et al. 1996; Haddix et al. 2003). The process of identifying, quantifying, and pricing these resources accurately requires a standardized methodology that systematically tracks and evaluates each resource consumed. We recommend the use of the “ingredients method,” also known as the “resource cost method.” This method was developed over 30 years ago; has been employed in hundreds of cost analyses of education, health, and criminal justice interventions (Belfield and Levin 2013; Levin and McEwan 2000); and can provide a solid foundation for meaningful comparisons of costs across programs. Numerous guides and examples are available, as well as tools to facilitate the process (Belfield and Levin 2013; Calculating the Costs of Child Welfare Services Workgroup 2013; Levin et al. 2006; Levin and McEwan 2000).

The ingredients method requires the evaluator to (1) describe the theory of change or logic model guiding the program; (2) identify specific resources used to implement the program; (3) value the cost of those resources (see section III); and then (4) estimate the program’s total, average, and marginal costs. This method includes estimating resource needs related to both the “day of implementation” and intervention infrastructure as well as variable and fixed costs (i.e., those that do and do not vary depending on the number served). This approach recognizes the importance of going beyond program budgets to include other measures of resource consumption that help illuminate how resources are used to achieve prevention goals (e.g., time logs, travel records, volunteer tracking, participant time, in-kind donation). Free tools for implementing this approach to cost analyses are available, including the online CostOut tool available through Columbia University’s Center for Benefit-Cost Studies of Education.

### Standard: Describe Comprehensively the Units and Resources Needed to Implement the Intervention, Disaggregated by Time

The ingredients method calls for a comprehensive assessment of all resources (e.g., labor, equipment, materials and supplies, space, travel) needed to carry out the program (Belfield and Levin 2013). Program descriptions, logic models, and the program’s theory of change can all be useful in specifying resources consumed by the intervention, when they are needed, and the activities in which they are consumed. Consulting these documents during the planning phase can help ensure that the cost analysis is comprehensive and aid in the prospective development of tools for estimating resource consumption.

When evaluating the cost of a program, resource costs are appropriately based on local prices and the context in which the program was implemented. Staff costs reflect actual local wage rates, and rent reflects local real estate market prices. Because these estimates may not generalize to other communities or future contexts with different economic conditions, it is essential to document both the amount of each resource (e.g., the number of full-time-equivalent bachelors-level social workers) and the prices of those units. Sometimes the prices for different resources will be followed across many years. For analytic purposes, all prices should be converted to a common year using an appropriate price index. We recommend using the year when the report is issued (“today’s dollars”).

Often the resources needed to implement a prevention program are consumed at different rates over a period of time (e.g., weeks, months, years). For example, capacity-building and training needs may be most intensive early on, whereas sustainability efforts are likely to increase over time. A robust understanding of when different resources will be needed (e.g., labor, materials, space, training vs. booster training) is key for program planning and project sustainability (Crowley et al. 2012; Shediac-Rizkallah and Bone 1998). Cash flow analyses can show the timing of costs as well as any inflows of money to support the intervention (e.g., fees charged to participants, revenue from funders to carry out the intervention). It may be helpful to consider when resources are acquired and paid for, as well as when they are actually consumed. Although the optimal time unit for disaggregation may vary across studies, researchers should at least estimate resources needed during the start-up phase of the project (i.e., the initial adoption and first implementation) and once regular operation of the project occurs (i.e., steady state). Whereas many impact evaluations will implement the program for a long enough period to assess such impacts, briefer pilot trials may be too short to determine such costs. In such cases, it is important to acknowledge such limitations when reporting cost estimates.

### Standard: Include Resources Consumed but not Paid for Directly

The costs of most program resources, such as wages provided to those employed to run the program, can be attributable to the program itself. However, some costs cannot. Examples may include volunteer time, travel time, and costs of participants, which, though not paid for by the program, do represent a resource that is used to make the program possible. Another example is indirect costs like overhead expenses, meeting space, utilities, and some forms of equipment that may be used by multiple programs run by a given provider, but which may be difficult to accurately attribute to any one program. (Husereau et al. 2013). It is important to understand and measure or estimate the amount of these resources consumed by the program and the types of activities in which they are used. The program may also result in additional costs that cannot be expressed easily in monetary terms, such as withdrawal symptoms, feelings of embarrassment, stigmatization, or psychological coercion. If there are theoretical reasons to expect that the program holds such costs, they should be acknowledged, even if not quantified or monetized.

### Standard: Resources Needed to Support Program Adoption, Implementation, Sustainability, and Monitoring Should Be Included in Cost Estimates

In a review of the field, we found that many cost estimates in prevention capture only the most immediate resource needs of programs (Hennessy et al. 2006; Lee et al. 2012). These “day of implementation” costs typically consider only the resource inputs most proximal to service delivery and often neglect crucial elements of the infrastructure that support program impact and were put in place during the evaluation trial. For instance, local knowledge about how to adopt and implement prevention programs with fidelity and high quality varies tremendously (Fagan and Mihalic 2003; Valente et al. 2007). Many programs now employ manualized training to teach program facilitators how to deliver a specific prevention curricula, but few train the managers overseeing those facilitators to ensure programs are delivered with quality or plan for program sustainability (Hawkins 2009; Johnson 2004; Perkins et al. 2011).

These skills are often taken for granted and assumed available in existing labor forces (Feinberg et al. 2008). The reality is that these skill sets may not exist within many local labor markets (Akerlund 2000; Savaya and Spiro 2011). To deliver prevention programs successfully and replicate the effectiveness of trials, local capacity must be built deliberately through training and technical assistance (Spoth and Greenberg 2011). Such capacity-building can require significant resources and, if not budgeted for, can undermine the entire prevention effort (Crowley et al. 2012; Scheirer and Dearing 2011). Although the field of prevention science has reached consensus that such capacity-building is essential for successful prevention (Spoth et al. 2013), only recently are some cost analyses reflecting the resources required for this work (Crowley et al. 2012; Kuklinski et al. 2012).

Infrastructure building occurs in four areas: adoption, implementation, sustainability capacity, and evaluation (Fagan and Mihalic 2003; Feinberg et al. 2004). *Adoption capacity* refers to a local community agency’s ability to select and be trained to deliver an evidence-based prevention program (Fagan and Mihalic 2003). This ability involves local capacity to understand the needs of the target population as well as the fit of the program (Chinman et al. 2005). Adoption capacity of evidence-based prevention strategies remains low and often requires considerable assistance (Ringwalt et al. 2002, 2008). Strategies such as Communities That Care pay explicit attention to developing this capacity (Brown et al. 2007; Feinberg et al. 2004).

*Implementation capacity* is the ability to deliver the program (generally from a manualized curriculum) and to ensure ongoing program quality (Berkel et al. 2010; Goodman et al. 1998). Many communities lack quality assurance systems that are compatible with prevention programs available in the current marketplace (Chinman et al. 2005). Developing these systems can take time but is key to ensuring that prevention services are delivered with fidelity in order to protect vulnerable populations (Chinman et al. 2008; Dunworth et al. 1999; Hawkins 1992; Spoth et al. 2004).

*Sustainability capacity* refers to the ability of a prevention effort to integrate the program into an ongoing service infrastructure and develop a robust funding stream to support ongoing programming (Gruen et al. 2008; Johnson 2004). This capacity requires training and technical assistance around fundraising, management of in-kind and volunteer resources, and in many cases expansion of programming in order to remain competitive (Akerlund 2000). Lastly, in order to assess both the implementation quality and whether prevention goals are being achieved, *evaluation capacity* is needed to *evaluate* proximal outcomes and provide feedback that ensures program adjustment and improvements are made as needed in the service of overall prevention objectives.

Each aspect of infrastructure involves resources and costs that may be substantial and variable across time (Crowley et al. 2012). All resources needed to build a successful prevention effort should be included in a comprehensive cost estimate of a program (Foster et al. 2007). Sustaining an intervention is a complex undertaking that may involve systemic issues far beyond a specific prevention program. The extent to which these activities are included in the analysis should be described. For those conducting cost analyses within an efficacy trial, long-term infrastructure needs may not be fully understood, and thus, resources cannot be assessed. Cost estimates developed in the efficacy stage may have important value for understanding training, implementation, and quality assurance costs and for guiding optimization of the program, but they should not be characterized as comparable to cost estimates of interventions that do include the entire set of infrastructure resources needed to disseminate and sustain the program.

Intervention development and program evaluation costs (e.g., conducting an efficacy or effectiveness trial) are also likely to be substantial but are conceptually distinct from the cost of implementing the program to achieve impact. As a result, these costs are not typically included in the cost analysis.

## Standards for Valuing Effects of Prevention Programs

The nature of human development is that early experiences in one domain can cascade across the lifespan into other domains (Dodge et al. 2008). Similarly, benefits of prevention may be distributed across domains (e.g., education, criminal justice, healthcare, and the labor force) and across the life course. This fact poses numerous challenges for estimating economic benefits, particularly those of covering all domains that carry an economic impact and converting behavioral outcomes to dollar values. In this section, we recommend standards that serve to reduce uncertainty and increase the rigor of estimates produced through benefit-cost analyses.

For the purposes of benefit-cost analysis, prevention benefits generally take the form of avoided costs or increased revenues that can be attributed to prevention program impacts (Crowley and Jones 2017; Karoly 2008; Vining and Weimer 2010). For example, prevention programs that reduce underage drinking could avoid costs associated with motor vehicle crashes. There are typically two steps in valuing the impact of a preventive intervention. First, for each individual in the intervention and control groups, (1) postintervention outcomes are measured during the postintervention time period, (2) converted to a monetary value on a common metric (e.g., today’s dollar values), and (3) summed. Second, the monetized benefit is computed as the difference in total monetary value between the members of the intervention group and the comparison control group and tested for statistical significance.

### Standard: Estimate Findings for Each Program Outcome Separately from Benefit Estimates and Describe the Context of the Evaluation

In prevention programs, there are many examples of outcomes that occur within the timeframe of the evaluation and may be directly valued, especially when follow-up spans into adolescence and adulthood. In a school setting, these outcomes may include educational counseling services, special education, or costs to process delinquent acts in the classroom. Valuation could derive from actual dollar values collected as part of the evaluation (e.g., medical costs pulled from hospital records) or from readily monetized outcomes (e.g., locally available court costs for processing a documented arrest). Observed outcomes during the course of the evaluation afford the opportunity for direct valuation. Some outcomes may have a direct monetary value (e.g., earnings), and some can be converted to a monetary value (e.g., annual costs of special education placement).

The monetary value applied to a particular behavior may be computed by any of several methods, and so the researcher should report the behavior change in natural units (e.g., effect size, odds ratio) separately from the valued benefit (i.e., monetized outcome) so that other researchers may recalculate benefits using alternative approaches. For instance, changes in public policy may affect the economic value from altering a behavior (e.g., the costs of a day in prison or the costs to provide an hour of special education can change with new laws).

Comprehensive economic valuation requires designing the evaluation to analyze all relevant domains of impact, both those measured during the evaluation as well as those not measurable in the timeframe of the evaluation. Program developers and evaluators should determine the course of intervention impact over time and assess impacts on persons in the comparison group. This measurement should include as much follow-up data collection as is feasible based on logistic and funding limitations. Measures of program impact that extend beyond participants should be based on the intervention’s logic model (e.g., effects on classroom peers or siblings).

The assignment of a monetary value to a behavior is made in a particular economic, social, and political context. Further, that context may vary across geographical regions or time. A program that serves to strengthen job skills may have differing economic benefits depending on how difficult it is to find a job. In a period of high employment, these skills may not be necessary to find a job, whereas in a recession, these skills may increase applicant competitiveness. A program that prevents psychiatric disorders with high utilization of welfare services may have differing economic benefits depending on the government’s funding for such services. For instance, a prevention program deemed cost-beneficial in a political environment of generous welfare services may become noncost-beneficial if a new government cuts funding for these services. By reporting behavior units separately from their estimated monetary value, researchers and policymakers will be able to compute updated benefit-cost ratios to estimate benefits for a programs offered in new contexts more easily. As described in section I, the evaluator should describe context in which estimates are made by noting the years covered, the geographical locale, and description of the participants (e.g., income, education background).

### Standard: Balance the Rigor of Direct Valuation of Outcomes with the Validity of Indirect Valuation in Contemporary Society

Whereas directly valuing outcomes provides the most straightforward approach to assessing benefits, sometimes logistic or analytic restrictions prevent direct valuation from occurring. Further, sometimes the outcomes that can be monetized directly do not represent the full impacts of the intervention. In such cases, evaluators often employ indirect valuation techniques to project program impacts into the future. Projecting impacts beyond those directly observed as part of the evaluation can enable a more accurate and complete estimate, but they also carry assumptions about the relations between present and future outcomes. Given the rich literature demonstrating the value of programs that yield long-term improvement in the developmental life course (Barnett and Frede 2010; Conti and Heckman 2013), there is justification for incorporating projections in economic evaluation (Slade and Becker 2014). While projection models allow for increased comprehensiveness in estimates, they also introduce increased uncertainty. This trade-off should be carefully considered when conducting economic evaluations.

One of the main approaches to indirectly valuing outcomes is known as shadow pricing. A shadow price is an estimate of an economic value when market-based values are unavailable (e.g., no market for buying and selling emotional regulation; Karoly 2008). Due to the long time horizon and multiple domains across which prevention outcomes unfold, such shadow price estimates can be used to provide estimates of the full impact of effective programs (Crowley 2014; Foster et al. 2003).

A classic example of shadow pricing is the value attached to receipt of a high school diploma in terms of increased income to the individual and tax revenue to the government associated with obtaining a degree, reduction in criminal activity as well as reduced reliance on publically financed healthcare and welfare services (Levin et al. 2006). Similarly, Cohen and Piquero (2008) and McCollister et al. (2010) provided valuations for different types of criminal behavior (e.g., aggravated assault, burglary, etc.). Corso et al. (2011) offered valuations for child maltreatment.

An economic evaluator will often face trade-offs in the decision to use indirect valuation for certain prevention outcomes. If one restricts indirect valuation only to areas in which direct impacts have previously been observed, important benefits may be neglected. Consider a program that increases a measure of kindergarten readiness. What is the best estimate of indirect valuation of this outcome? Reliable empirical evidence shows that this indicator predicts third-grade special education placements, which has immediate economic value. It also predicts high school graduation, albeit with less precision, and a rational case can be made that it would predict ultimate employment. What valuation should be used? At issue is the extent to which the cost of special education or extent of gains in high school graduation is attributable to prevention program impacts on kindergarten readiness. A reasonable case could be made for both a more conservative valuation (e.g., restricting the impact to only savings from a reduction in utilizing third-grade special education) or a more liberal valuation (e.g., changes in school readiness related to changes in graduation). In such a scenario, evaluators need to justify their decision and test the impact of choosing upper- and lower-bound estimates. Such tests, known as sensitivity analyses, are covered in section V (handling estimate uncertainty).

The quality and consensus on shadow prices can vary by substantive area. As such, it is important to consider carefully the utility of using shadow prices given the purpose of the evaluations. Sometimes the shadow price is imperfect but may currently be the best estimate and appropriate for indirect valuation. Sometimes an estimate is only appropriate for projections in certain circumstances and should not be generalized (e.g., the economic value of graduating from high school is not equal to the value of receiving a GED). Sometimes a shadow price estimate is outdated and should be replaced with newer estimates (e.g., new estimates are frequently produced around the cost of different crimes). For instance, a somewhat controversial source of benefits in some prevention programs is quality-of-life improvements, as indexed by measures such as a disability-adjusted life year (DALY). The DALY is a measure of the burden of disease or disorder, computed by summing the value of life years lost prematurely plus the proportion of the quality of life lost each year while still surviving (both discounted to present value). These quantitative figures are based on qualitative judgments of the value of life and the suffering experienced by surviving persons afflicted with a disorder, automobile crash, or other adverse outcome—typically judged by stakeholders or evaluated from historical jury awards. As an example, based on surveys with caregivers and pediatricians, Miller et al. (2014) estimated the savings for avoiding one case of abusive head trauma to a child aged 0 to 5 as $180,407 per DALY. Using an estimate that abusive head trauma leads to an average of 29.9 life years lost per case, they computed the value of one child death due to abusive head trauma as $6.6 million, with a 95% confidence interval of $2.3 to $9.8 million. A program that prevents one case of this outcome can be credited with averting $6.6 million. Similar estimates have been used to derive the negative monetary value of the life-long behavior of a chronic criminal who causes harm to victims who suffer DALYs (Cohen et al. 2004). Because the computation of shadow prices may raise questions or may not generalize, in addition to conducting sensitivity analyses (section V), we recommend reporting benefit estimates that are disaggregated by the beneficiary (e.g., taxpayer, recipient, and victim).

### Standard: Consider Outcomes with Negative Monetary Values as Negative Benefits Rather than as Part of Program Costs

Sometimes an outcome variable will carry both positive and negative monetary benefits. An example is college attendance. As an outcome of a prevention program, college attendance carries immediate negative benefits (e.g., payment of tuition, lower income while a student), but also longer-term positive benefits in later income and better health and overall welfare. Another example is employment. As an outcome of a skills-training program for single mothers, employment brings negative benefits in the form of increased childcare and transportation expenditures and positive benefits in the form of higher income. Some analyses place the negative benefits on the cost side—others, on the benefits side. We suggest that the field adopts a standard for placing all monetary values that accrue as a direct part of the intervention and during the intervention period into the cost “bin” and all monetary values that accrue after the intervention ends into the benefits “bin,” acknowledging that some outcomes will have negative value. Doing so functionally deducts the negative outcomes from the positive outcomes instead of inflating the cost of the program. We suggest this standard because it brings clarity to these terms and will reduce confusion in interpreting results. Specifically, costs are average monetary values that are assumed to occur for every participant by definition of being assigned to an intervention. Benefits are monetary values that follow from outcomes that may or may not occur as a function of the experience of being assigned to intervention, with a higher or lower probability for recipients. Like the college attendance example above, numerous other outcomes carry both positive and negative monetary values that are best summarized as a single net value rather than a negative cost and a positive benefit.

## Standards for Summary Metrics

The final step of modeling the costs and benefits of an intervention is to compute a net-benefit score for intervention recipients (often calculated as the differences between the benefits and costs after making any needed adjustments for inflation and discounting). Yet, costs and benefits of prevention are not static. Thus, to increase the clarity and utility of the information provided, it is important to calculate and report a broad spectrum of metrics that describe program costs and benefits from multiple perspectives. Constructing these metrics differs from simply reporting a coefficient and *p* value. For example, cost estimates can be disaggregated both according to different program elements or phases (e.g., adoption, implementation, and sustainability) and according to who bears the costs (e.g., school system or healthcare system). Similarly, benefits can be disaggregated according to source (e.g., crime, graduation rates, and productivity) and to beneficiary (e.g., crime victims, justice system, and society). The metrics reported ultimately summarize the evaluation’s findings. When well done, they provide an evaluation that is transparent and highly useful for program planning and budgeting. When done poorly, the metrics reported can obscure important issues and misinform decisionmakers.

### Standard: Estimate All Costs and Benefits in Current Monetary Units or in Monetary Units for the Most Recent Year Available

One of the overarching considerations to be taken into account when constructing metrics is to characterize accurately the timing of the intervention’s benefits and costs. In evaluations of preventive interventions that span many years, this is of particular concern as the costs and benefits are subject to inflation. Inflation, the general rate at which prices for goods and services are rising, can be accounted for by simple conversion using a relevant consumer price index. For those unfamiliar, a multitude of free software and guides are available. Although the accuracy of an analysis will not be compromised by expressing benefits and costs in another year, using current dollars gives decisionmakers a better sense of the practical size of benefits and costs in the context of current purchasing power.

### Standard: Estimate Current Values for Benefits and Costs That Accrue over Time by Selecting and Reporting a Reputable Discount Rate

Ignoring inflation, a dollar of future economic benefits is worth less than a dollar of current program costs (i.e., a dollar today is worth less than a dollar next year; Boardman 2011). To account for this phenomenon, economic evaluations generally discount future costs and benefits at a rate of between 3 and 7% each year past the first year of implementation (Claxton et al. 2006; Gravelle et al. 2007; OMB 1992). Depending on the length of a program and the period of evaluation follow-up (e.g., 30 years), an annual discount rate can have meaningful impacts on the reported metrics. In such cases, the effect of using alternative different discount rates on summary metrics should be reported.

### Standard: Estimate and Report the Total, per-Participant Average, and Marginal Costs of the Program

The principal summary metrics for describing an intervention’s costs are the total, average, and marginal costs of the program. The total cost is the sum of all ingredients consumed implementing and running the program during the implementation trial. If the study has multiple arms, it is necessary to assess and report costs separately for each arm of the intervention. When an intervention is conducted at multiple sites, researchers need to consider carefully whether to aggregate costs across sites or to report costs on a site basis. The per-participant average cost is the total cost divided by the number of participants assigned to the program. Although actual costs often vary across participants (for example, some participants may fail to show up for some sessions), this variation is usually due to self-selection. In these cases, it is difficult to identify causal effects of the dose of participation on an outcome. Because it is often difficult to predict self-selection patterns in future implementations, it is problematic to assume that different cost estimates can be assigned to different groups in a reliable way. When selection is correlated with a group characteristic such as gender or income, it may make the assignment of different cost estimates to different groups meaningful. For example, perhaps females, on average, show up for more individual counseling sessions in a voluntary prevention program than do males, leading the researcher to estimate the average program cost to be higher for females than males.

Sometimes when researchers disaggregate the average cost according to whether an assigned participant actually participates, they compute two different impact estimates—one for the total sample (called intent to treat [ITT]) and one for the participating subsample (called treatment on the treated [TOT]). The validity of the TOT estimate depends on participation being exogenous to any characteristic of the assigned participants (e.g., perhaps after random assignment, a computer glitch prevented the interventionists from contacting a random subgroup of assigned participants who never got the opportunity to participate).

The marginal cost of a program is the cost of adding participants. Consumers of reports of program cost estimates often want to extrapolate the estimated cost for a future implementation with a different sample size. Extrapolations usually cannot be made linearly because some costs are fixed and some are variable. Fixed costs are required for a future implementation no matter how individuals participate, at least within a certain range. Variable costs depend on the number of participants. For example, perhaps a group intervention has a fixed cost for the group leader’s salary and variable costs for food and transportation for each participant. Adding another participant increases the fixed cost by nothing and the variable cost by the food and transportation cost for that participant. When such nonlinearities occur, the average and marginal cost of a program will differ. We recommend disaggregating costs according to fixed and variable costs and also reporting the marginal cost of serving an additional participant.

### Standard: When Applying Benefits Across Multiple Outcomes to Generate Total Economic Values, Avoid Double-Counting Economic Impact

Economic evaluators typically identify program impacts, estimate an economic benefit for impacts that are monetizable, and then sum these values to reach a total benefit. When summing benefits, it is essential that evaluators not double-count benefits (Drummond et al. 1993; Zerbe et al. 2010a, b). Double-counting can occur when multiple program outcomes lead to the same type of benefit (e.g., healthcare utilization, educational attainment). Indeed, in many cases, prevention programs target multiple competencies that may combine and interact in their influence on future monetizable outcomes. Specifically, the equifinality of many developmental processes (i.e., the phenomenon that many different pathways and processes can lead to the same outcome) makes it unclear whether benefits are overlapping, additive, or interactive, which can make projections difficult (Cicchetti and Rogosch 2009). For example, a prevention program may lead to lower rates of substance abuse disorder and arrests among participants. Improvements to each condition typically would lead to improvements in employment. If the employment effects from the two impacts are not fully independent, summing will overstate the economic benefits that can be attributed to the prevention program.

Evaluations that employ indirect valuation approaches are particularly at risk for inaccuracies. For instance, evaluators of a program that reduces substance abuse and delinquent behavior typically should not project savings from reduced incarceration, reduced healthcare costs, and increased employment for each outcome and then simply add the savings together (Lee et al. 2012). Instead, they must adjust estimates to avoid projection overlap. One approach is to employ a series of “trumping rules” that isolate developmental pathways to ensure no double-counting occurs (e.g., Aos et al. 2011). One example would be to use the most proximal predictor in terms of measurement to determine which should be the primary predictor. Alternatively, one may choose to use the predictor with the strongest relation to the outcome of interest. Another option may be the predictor that best maps onto the intervention’s logic model. For instance, in a program that reduces delinquency and substance abuse, only the delinquency effect might be used to project incarceration, and only the substance abuse effect might be used to project health benefits. Trumping rules typically provide more conservative estimates of program benefits and overall economic impacts, which can be useful to decisionmakers. The need for such rules is one of the limitations of indirect valuation approaches. Evaluators should make sure that any trumping rules are clearly defined and carefully described.

### Standard: Use the Net Present Value with a Confidence Interval as the Principle Summary Metrics of Benefit-Cost Analyses

An intervention’s net present value (NPV) is the difference between present value benefits and present value costs (Boardman 2011; Levin and McEwan 2001; Zerbe et al. 2010a, b; Zerbe and Dively 1997). It is the preferred summary metric of benefit-cost analyses because it captures two important pieces of information. First, when positive, it indicates that the program being evaluated can be cost-beneficial. Second, the size of the NPV captures the net social benefits to society in dollar terms when the societal perspective is used to frame the analysis, something that the alternative summary metrics described below do not achieve. If a more limited perspective (e.g., governmental) is applied, the NPV will reflect the net benefit from that perspective.

The NPV can be estimated on a per-participant basis or for the entire sample for which the BCA was conducted. The per-participant value is easier to grasp and usually the level at which model testing and statistical significance are computed, but the sample-based value provides a more complete picture of the overall loss or gain resulting from the program.

### Standard: Describe the Advantages and Limitations of Any Additional Summary Metrics That Are Included in the Evaluation. Some Metrics Should Be Used Only when Certain Conditions Are Met

In addition to the NPV, BCA results can be summarized in terms of the benefit-cost ratio, payback period, return on investment (ROI), internal rate of return (IRR), and investment risk. Each has potential drawbacks that should be understood and communicated alongside results where applicable (Boardman 2011; Levin and McEwan 2001; Zerbe et al. 2010a, b; Zerbe and Dively 1997).

The benefit-cost ratio (BCR), which divides present value benefits by present value costs, has intuitive appeal as a summary of the “dollar returned per dollar invested” heuristic. However, because it is a ratio, the BCR does not provide information about the magnitude of present value benefits or present value costs. As a hypothetical example, a high BCR could follow from a program that returns $1000 in benefits per $100 in costs or $10 in benefits per $1 in costs. In both cases, the BCR is $1:$10, but the former program leads to a far greater welfare gain. For this reason, the BCR is most useful when offered in conjunction with the NPV.

The payback period, breakeven point, return on investment, and IRR can provide useful supplemental information under certain conditions. The payback period summarizes the point at which cumulative discounted benefits equal cumulative discounted costs. This information may be sought by stakeholders wanting to understand the time horizon over which program investments break even. The concept is most relevant for programs and policies that require investments for a single, discrete period of time. The premise of many prevention programs is that benefits will continue after the termination of the intervention, although many programs experience fadeout of impacts over time. In a hypothetical example, a program costs $100 per individual and causes $20 in discounted annual benefits for the foreseeable future. The breakeven point, that is, the date at which the net benefits equal the costs, would be just over 5 years, depending on the rate of inflation and the discount rate applied ($100 Cost < 5+ Years * $20).

The payback period can be applied to programs for which benefits are negative for a temporary period. Temporary negative benefits in this case refer to expenditures incurred outside of program costs that are related to a program outcome. One example is a middle school program designed to prevent high school dropout that leads to temporary negative benefits when more program students remain enrolled in high school. The logic model of this program is that ultimately the benefits will become positive through enhanced employment and other outcomes. A second example is a prevention program targeting a high-risk sample of youth and their families that leads to temporary negative benefits when participating families ask for, or are referred to, mental health or healthcare services they otherwise would not receive. A program with temporary negative benefits could achieve a positive cumulative net benefit at a later point in time. These cases illustrate that the payback period should be estimated and reported with care, particularly when there is a period of negative benefits.

The IRR is the discount rate at which the NPV is 0 and can be interpreted as an annualized effective rate of return on an investment. An IRR larger than the discount rate that reflects the opportunity cost of the investment in the program indicates a favorable investment. The IRR is problematic under the same conditions that lead to problems for the payback period. Under these conditions, there are multiple possible IRRs, resulting in a lack of clarity about the “true” rate of return. In such cases, the IRR should be used with caution.

Program costs and benefits can be estimated on a per-participant basis for the entire sample on whom the estimates were based, or both. Each choice offers valuable information about the economic impact of the implemented program, as shown in Table 2. For example, the parenting program in Table 2 reaches fewer participants and costs more per participant, but it costs less overall to implement than the school-based program. It also has a higher NPV on a per-participant basis but a lower NPV overall. Finally, it has a higher BCR than the school-based program. Though the school-based program has a lower BCR, it reaches more students and, as noted, has a greater overall NPV. However, it is costlier to implement.

**Table 2:** Total and per-participant metrics both provide useful information

Program	Participants	Per participant			Total program			BCR
		Costs	Benefits	NPV	Costs	Benefits	NPV	
Parenting program	100	$75	$300	$225	$7500	$30,000	$22,500	4.0
School-based program	1000	$40	$80	$40	$40,000	$80,000	$40,000	2.0

Presenting information on a total and per-participant basis helps illustrate why a program’s net present benefit should not be the ultimate driver of resource allocation decisions. In the example above, both programs pass the benefit-cost test and, from a BCA perspective, reflect investments that should be “on the table” from an economic perspective. Both could have a place in a portfolio of investments devised to achieve an overall set of goals.

## Standards for Handling Estimate Uncertainty

Because each measurement at each step of an economic analysis is made with some degree of unreliability (just as each measurement in an impact evaluation has unreliability), the final estimate of economic impact will have some uncertainty. Standards are needed for estimating, resolving, and reporting that uncertainty.

### Standard: Test the Implications of Uncertainty in Estimates and Report the Manner in Which Uncertainty Is Handled

Intervention cost and benefit estimates are based on a variety of assumptions, and the impact of uncertainty in those assumptions should be modeled (Crowley et al. 2013; Foster et al. 2007; Haddix et al. 2003). A deep understanding of factors that lead to variation in estimates allows more robust projections. Best practice is to provide estimates within a confidence interval and to avoid point estimates without confidence intervals whenever possible (Haddix et al. 2003; McCall and Green 2004). Sensitivity analyses should also be employed to evaluate variability in assumptions systematically (Briggs et al. 1994).

Uncertainty in economic evaluations can generally be traced back to the measurement of resource use, the measurement of outcomes, and the application of monetary values to outcomes, but additional sources of uncertainty from analytic decisions and assumptions also influence cost and benefit estimates (Boardman 2011; Levin and McEwan 2001; Welsh 2014; Zerbe et al. 2010a, b; Zerbe and Dively 1997). The challenge for analysts is to acknowledge uncertainty while maintaining a clear statement about the findings.

Including point estimates or expected values of costs and benefits as well as their confidence intervals balances a “bottom line” best estimate with a range that conveys to decisionmakers, in general terms, the precision of an estimate and its likely stability in subsequent implementations. Confidence intervals can be estimated even when the economic evaluation is based on a single implementation through the use of Monte Carlo analysis, or other methods such as bootstrapping (Boardman 2011). Monte Carlo analysis software can perform large numbers of cost analysis and BCA simulations (e.g., 100, 1000, 10,000) that randomly vary multiple parameters used in the analysis based on their sampling distributions, leading ultimately to variability in cost analysis and BCA results and the necessary foundation for establishing confidence intervals. Oracle’s Crystal Ball and Palisade’s @Risk are two relatively easy-to-use software packages that perform Monte Carlo analysis. The approach enables the estimation of confidence intervals around various metrics used in the analysis as well as total benefit and cost estimates.

Analysts may also want to understand the sensitivity of results to variability in a single or set of judiciously chosen parameters, such as intervention effect size(s), costs and resource use, the discount rate, the time horizon over which benefits are projected, and other factors of import to a given economic evaluation and/or prevention program implementation, such as decisions to account for equity concerns (e.g., lower pay for women and minority populations) through weighting or other procedures (see National Academy of Medicine et al. 2016). Although the results of these focused analyses may be informative, they yield only a partial picture of the implications of uncertainty, in that they do not consider variability in the full range of parameters that influence the cost analysis and/or BCA estimate, which should be acknowledged when these methods are used. The rationale for testing such variations should be clearly articulated and contrasted to the original estimate(s) of interest.

## Standards for Reporting Findings from Economic Evaluations

The process and manner of reporting economic evaluation results is highly dependent on the evaluation conducted, but several overarching approaches should be followed to enhance the comparability of studies both within and outside prevention science. The following standards address best practices for both conducting and reporting cost analyses and benefit-cost analyses for prevention. Every effort was made to balance the burden from reporting with the need for a clear record of how the evaluation was conducted. For researchers publishing in shorter format outlets, we highly recommend the use of appendices whenever possible.

### Standard: the Principle of Transparency Should Guide the Reporting of Economic Evaluation Results

Transparent reporting increases accountability, contributes to the credibility of economic evaluation findings, and facilitates comparisons across studies. Economic evaluations of prevention need to be reported with enough detail that readers are aware of the strengths and limitations of the analysis and have sufficient information to support decision-making. As discussed, information from economic evaluations can be useful to a variety of stakeholders, including researchers, program developers, program administrators, policy analysts, advocates, intermediaries, and those charged with making decisions about which programs to fund given limited societal resources. Although each of these groups may have different reasons for seeking results from an economic evaluation, all will benefit from clear reporting that provides sufficient detail about the methods and assumptions underlying the results to support the validity of the findings.

After reading an economic evaluation report, readers should understand the conditions under which results are likely to be replicated, the extent to which findings are likely to be generalizable, and how they inform the decision at hand. The major elements to be communicated in the report are summarized in Table 3 (Karoly 2008; National Academy of Medicine et al. 2016). This information is particularly important when alternative program options are being considered so that meaningful comparisons can be made. For example, if one economic evaluation includes both education and criminal justice benefits, while another includes only criminal justice benefits, the estimates may not be comparable. If the respective reports describing the results fail to mention what exactly is included in the benefits estimates, incorrect inferences will be drawn.

**Table 3:** Key information for reporting economic evaluations

Topic	Essential information to communicate in a report	Standards section
Economic evaluation frame	• Research, policy, budgeting, and/or decision-making context in which the economic evaluation was sought • Specific empirical question to be addressed in economic evaluation • Analytic perspective, e.g., societal, government • Time period over which costs and/or benefits will be estimated	I.1, I.4
Intervention Description	• Intervention goal(s), theory of change, and/or logic model • Population served, delivery setting, major intervention components • Location, time, key features of context in which intervention was or will be delivered • Comparison condition, e.g., no program, alternative program	I.2
Intervention impacts^a^	• Research design that yielded impacts, including any limitations on ability to draw causal inference • Key characteristics of treatment and comparison groups, including any differences and how they were controlled for • Length of follow-up and attrition rates for each condition • Quality and sources of data and measures used • Summary of impacts, including magnitude, significant and nonsignificant findings, significance levels, standard errors or standard deviations, methods of estimation • Description of plausible additional impacts that were not measured, why they were not measured, and implications for the economic evaluation • Any limitations to generalizability, internal and external validity of impacts	I.3
Cost estimates	• Whether analysis is being conducted prospectively or retrospectively • Method for estimating costs, e.g., ingredients method • Cost categories included, e.g., labor, equipment, materials and supplies, office space, travel; any costs that were excluded and reasons for exclusion • Method for costing volunteer time and other donated resources, overhead, and other resources not paid for directly • Scope of costs included, e.g., adoption, implementation, sustainability, training and technical assistance, including rationale for any exclusions • Source of resource and unit cost or price data • Any limitations to generalizability and validity of resource, price, and cost data	II.1–II.5
Benefits estimates^a^	• Summary of impacts included in the benefit-cost analysis, those not included, and rationale for inclusion or exclusion • For each impact, whether benefit was estimated directly or indirectly • Method and model for estimating benefits from each included impact • Length of time over which benefits were estimated or projected • Any negative benefits and how they were handled • Sources of data used to derive benefits estimates, including support for any shadow prices used • Implications of impacts that were not monetized on benefits estimates • Any limitations to generalizability and validity of benefits estimates, including modeling as well as data sources used in the analysis	III.1–III.3
Discounting and inflation	• Year in which constant dollars are reported • Inflation indices used in costs and/or benefits analysis • Discount rate, including range used in sensitivity analysis • Time or age discounted to, e.g., participant age, program start	IV.1, IV.2
Summary metrics	• Total, per-participant average, and marginal costs of the intervention expressed in constant discounted dollars • Total and per-participant benefits from the intervention, including description of how potential double counting was handled, expressed in constant discounted dollars in the same base year as costs^a^ • Net present value in constant discounted dollars (total and per participant)^a^ • Additional summary metrics, e.g., benefit-cost ratio, payback period, internal rate of return and any relevant limitations^a^ • Relevant disaggregated costs, e.g., fixed and variable; costs by relevant time period; costs by stakeholder; capacity-building, implementation, sustainability costs; labor, supplies, space, travel, overhead costs • Relevant disaggregated benefits, e.g., according to beneficiary; by impact; by sector^a^ • Standard errors and confidence intervals associated with each metric	IV.3–IV.5
Handling of uncertainty	• Method used to evaluate implications of uncertainty, e.g., Monte Carlo, bootstrapping, sensitivity to changes in key parameters • Implications for summary metrics and analytic conclusions	V.1
Conclusions	• Statement(s) relating analysis findings to original question • Generalizability, replicability, external validity, and limitations of conclusions reached	VI.1

^a^These elements are not required for a cost analysis

Transparency in reporting is critical because all estimates involve assumptions, decision rules, and uncertainty. We recommend separate reporting of (a) program costs, (b) prevention impacts on behavior, (c) prevention impacts on economic benefits (without regard to program costs), and (d) the intervention’s net benefits as appropriate. Best practices for transparent reporting include comprehensive enumeration of the assumptions and decision rules used in analysis, models and sources of price information for valuing benefits (i.e., direct valuation), quality of information used to project benefits (i.e., indirect valuation), and description of the conditions under which results are likely to replicate. Finally, to be useful to policymakers, program administrators, and other consumers, reporting should be clear, concise, and easy to understand.

### Standard: Use a Two-Step Reporting Process That Summarizes the Most Essential Features and Results of an Evaluation in a Table or Brief Report and Offers Supporting Technical Detail Elsewhere

A challenge in reporting economic evaluation results is maintaining transparency while avoiding reporting complexity that can ultimately lessen the comprehension of findings. Achieving this balance can be difficult due to the many decisions and factors driving economic evaluation, particularly BCA, some of which are highly technical. We recommend implementing a two-tiered reporting system that includes a consumer-focused summary accompanied by a technical description (e.g., included as an appendix) that details the modeling and assumptions made to estimate costs and benefits. The objective is to offer cost analyses and BCA bottom lines that succinctly address the questions driving the analysis in a summary report, including concise presentation of the elements in Table 3, with reference to a source that provides more detailed information on the validity of summary estimates. The best reporting format will vary with the audience. Decisionmakers, particular budget writers and funders, may want technical detail completely separate from a brief report, which may be limited to a single page. Academic journals are likely to want many of the technical details summarized within the paper and will relegate the more nuanced discussion of decisions to online supplemental materials. Ultimately, standards of research reporting require sufficient technical detail so that another researcher could replicate the methods and reach the same findings. Supplemental documentation, accessible online, is likely to be necessary to report the methods and assumptions driving many economic evaluations of preventive interventions, particularly those relying on complex projection models.

### Standard: When Monte Carlo Analysis Is Performed, Present a Histogram of the NPV Distribution as well as the Percentage of Simulations That Return a Positive NPV

**Fig. 1:**
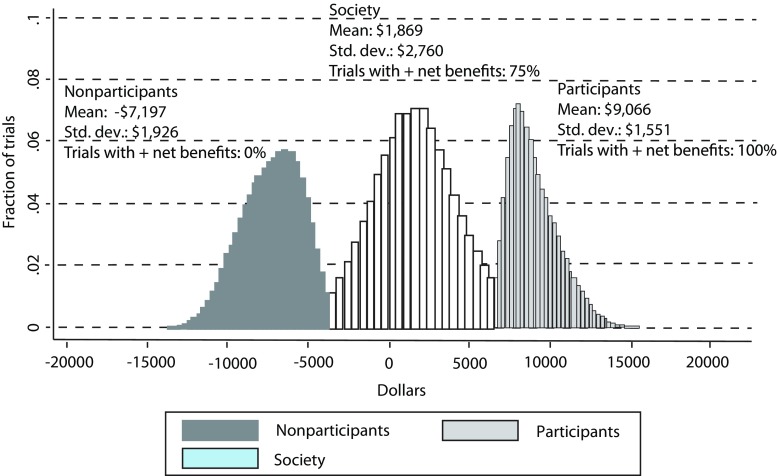
Histogram of net present values generated from Monte Carlo analysis

As described above, sensitivity analyses using Monte Carlo methods involve many simulations of the likely NPV. The predicted NPV for each simulation can be represented in a histogram that gives a sense of the distribution. Further, the proportion of simulations that achieve a positive NPV can be calculated. These two pieces of information capture in a simple straightforward way the implications of uncertainty for the NPV of a BCA and can communicate information to potential funders about the riskiness of investing in a program. As shown in Fig. 1, the histogram (Carlson et al. 2011, reprinted with permission) conveys pictorially the range of NPVs produced by the Monte Carlo analysis as well as the frequency with which values occur. This illustration highlights the degree of precision in the expected value of the NPV and provides information about the extent to which negative NPVs, or unfavorable results, were found. It also shows the distributions of NPVs for the participants and nonparticipants, which are typically of great interest to policymakers. The percentage of positive NPVs summarizes how likely it is that a program would be cost-beneficial when replicated, given multiple sources of uncertainty that could influence results in subsequent implementation.

## Limitations of Economic Evaluations Within Prevention Science

The proposed standards aim to address the ability of economic evaluations to offer consistent, high-quality, transparent estimates while also providing the flexibility essential to evaluating the many forms of prevention. The MAPS III Committee believes that it is important to recognize the limitations of economic evaluation methods—even when conducted in a high-quality manner consistent with the above standards (e.g., Caulkins 2014). Specifically, findings from an economic evaluation are an important factor, but they should not be the sole determinant of programmatic and funding decisions, for reasons of both internal and external validity. First, as with other evaluation methods, the internal validity of any specific method has limitations. The evaluator makes assumptions (which should be made explicit) and then judgments about which impacts to quantify and monetize as well as other assumptions needed to conduct the analysis. Time and resource constraints can prevent investigation of all possible benefits and costs. Some effects may be inherently nonquantifiable, or impossible to assess in financial terms, yet considered crucial to a program’s success or political viability.

Second, the figures produced in economic evaluations of a prevention program that had been implemented at one time in one context may not generalize to other times and contexts. Prevention program costs depend on wages, other resources, and economies of scale that vary. Benefits depend on the cost of services avoided, income tax rates for participants’ employment, overall economic and demographic conditions, and government context of social services funding, all of which vary over time and locale. For example, as contraceptive technology changes, the costs of pregnancy prevention programs will change. The current report addresses standards in the economic evaluation of a program as it was implemented; estimating costs and benefits for a program that may be implemented at a future time and context should be done with appropriate caution.

Economic evaluation is never a substitute for democratic and administrative decision-making (Drummond and Jefferson 1996; Vining and Weimer 2010). The funding decision could depend on the benefits and costs of another program with similar objectives, the total budget available for funding such programs, and contextual priorities. Policy decisions must take into account moral, ethical, and political factors (Zerbe 2005; Zerbe et al. 2010a, b). Legislators and other public officials must allocate scarce public resources among many competing uses, such as reducing crime or risky sexual behavior, protecting the environment, supporting research, providing economic security, improving infrastructure, and improving health. Choices about how to allocate resources inherently embody judgments about relative benefits and costs. Economic evaluation seeks to provide an objective framework to make the basis of such choices explicit, so that stakeholders can better weigh the economic trade-offs. When carefully executed with attention to the findings’ sensitivity to different assumptions, these techniques can improve the basis on which funding decisions for prevention programs rest.

## Research Priorities in Economic Evaluation of Prevention

Recognizing these limitations, and alongside the standards outlined above, the MAPS III Committee offers a set of research priorities developed to enhance the quality and practice of economic evaluations in prevention science.

## Increase Focus on the Development of Shadow Prices

As noted, the long-term nature of typical returns to prevention dictates that benefits often must be estimated based in part on indirect valuation using shadow pricing. The quality of economic evaluations will improve as the stock of available shadow prices increases for important child developmental milestones and indicators. Here is where a synergy could emerge between developmental psychologists and economists. Life course developmental psychologists commonly estimate the relation between early behavioral patterns (e.g., conduct problems) or experience (e.g., child abuse) and later outcomes that carry monetary value (e.g., incarceration, substance use disorder), but these relations are typically reported in basic science journals as regression coefficients rather than shadow prices. High-quality estimates of the shadow prices of childhood behaviors and experiences could enhance economic evaluations of prevention. In particular, efforts to strengthen our ability to project future economic outcomes accurately are of particular importance. Such work likely necessitates employing models to strengthen the ability to draw causal inferences about the relation between proximal and distal outcomes. Such shadow prices will need to be updated as the social context changes.

An illustrative example related to prevention in early life might consider the economic value attached to school readiness as an outcome measure. This outcome is not directly measured in dollars, nor is there a natural direct valuation method. An indirect approach would utilize empirical research that establishes a causal link between observed readiness and another outcome that can be more readily valued in monetary terms. Research could link school readiness to later school performance (e.g., high school graduation) and then link school performance to labor market earnings, which may be valued. Such published shadow price estimates can provide important information on outcomes commonly targeted in prevention.

Shadow prices for behaviors targeted by prevention programs also may inform the upper bounds of what an intervention may cost to still be cost-beneficial. They may drive prevention scientists to develop new interventions that target behaviors and experiences whose benefits have particularly high shadow prices, and with parameters for creating prevention programs that are likely (or not) to be cost-beneficial if effective. For example, knowing that one case of career criminality has a shadow price of several million dollars (Cohen et al. 2004) could embolden prevention developers to design seemingly expensive interventions that might turn out to be cost-beneficial.

## Evaluate Program Costs Within Different Public Systems

Many preventive interventions are administered within the context of another government service, such as public schools. Although costing such interventions may seem obvious by identifying the budget allocated for the intervention, implementation of these interventions often relies on existing personnel, space, and services and, therefore, may require that resources be shifted rather than new dollars allocated. As another example, a cost analysis of an after-school program that takes place between 4 and 6 p.m. in otherwise unoccupied school rooms that have no potential for use for another purpose might not include space costs because the “marginal” cost of using these rooms for this particular implementation of the program is almost nil (save for electricity, etc.). Prorating the space costs based on proportional use would overestimate actual costs. Research is needed to understand the implications of various models of costing prevention programs when implemented in other service contexts.

## Identify Moderating Factors of Economic Impact

A prevention program may have different impacts on subgroups of intervention targets. Such heterogeneity in intervention response is common in prevention programs. For example, a universal preschool program may have stronger economic impacts on lower-income children than middle-income children. Understanding these patterns can guide policy-making, future prevention design for those groups for whom cost-beneficial interventions have not yet been identified, and funding plans. However, statistical testing on the moderation of economic benefit is still relatively crude. Furthermore, the detection of moderation within an implementation that included multiple groups does not imply that the positively affected group would continue to enjoy benefits in a future implementation that excludes unaffected groups. It may be that implementation is less costly per child in the larger context or that the positive impact for one group is contingent on inclusion of the unaffected group, particularly in an implementation context in which intervention is administered in a group format. These issues call for new empirical inquiry, specifically, a more focused effort to understand the economic implications of heterogeneity in response—particularly in universal programs. Of key importance is to understand whether any detected differential responsiveness is likely to change not only the intervention’s effectiveness but also its costs, benefits, and overall return-on-investment. Such analyses are key to assisting policymakers, budget makers, and program planners in making the best use of limited resources.

## Contrast Economic Impact of Change Induced by Prevention Versus Natural Environmental Processes

Research is needed to understand the life course of behavior and economic benefit that occurs as a result of preventive intervention in contrast to the same behavior that occurs naturally, without intervention. Consider the example of increasing high school graduation, estimated to have an economic benefit of more than $200,000 per graduate across the lifespan (Levin et al. 2006). However, a high school diploma that is earned through the GED does not carry those economic returns (Heckman and Mosso 2014). One could imagine a prevention program that facilitates high school graduation without the life skills development that naturally occurs during the normative path through high school. In such a case, would the economic returns that have been estimated from shadow pricing studies be realized? Longitudinal follow-up studies after preventive intervention trials end could illuminate the mechanisms through which economic benefits derive from specific behaviors and achievements.

## Committee Note

Mapping Advances in Prevention Science (MAPS) are multidisciplinary task forces commissioned by the Society for Prevention Research and funded by the National Institute on Drug Abuse. They are designed to address prevention research and practice in areas deemed especially important to advancing the state of the field. The MAPS I task force focused on the role of biological factors in prevention research, and MAPS II focused on type 2 translational research. The current MAPS III focuses on the economic costs and benefits of preventive interventions and policies. The charge of the MAPS III task force was to (1) provide guidance regarding current standards and best practices in the economic evaluation of preventive interventions and policies, (2) recommend actions to increase the field’s capacity to conduct and report high-quality economic evaluations, and (3) identify research gaps and policy needs specific to economic evaluation of prevention. This report summarizes the findings and recommendations of the MAPS III Task Force.

The MAPS III report and recommendations are the result of an iterative process, incorporating input from the members of the task force who were selected to represent diverse disciplines, areas of expertise, and perspectives. Members sought input from representatives of key federal agencies (e.g., National Institute on Drug Abuse, National Institute of Alcoholism and Alcohol Abuse, and the National Academy of Medicine), private foundations (e.g., MacArthur, Pew, Robert Wood Johnson), policy institutes (e.g., Brookings, RAND), the interdisciplinary Prevention Economic Planning and Research Network (PEPR), and attendees of annual conferences for both the Society for Prevention Research and the Society for Benefit-Cost Analysis. After deliberation, the task force reached consensus about standards of quality that should be endorsed in evaluating the economic costs and benefits of prevention interventions and policies. The current document summarizes those standards.

## Electronic Supplementary Material

ESM 1 (DOCX 14 kb)
