# Supplementary material for: Standards of Evidence for Conducting and Reporting Economic Evaluations in Prevention Science
Source: Prev Sci. 2018 Feb 12;19(3):366–90. doi: 10.1007/s11121-017-0858-1 (PMC5869868; doi:10.1007/s11121-017-0858-1)
Supplement: Supplementary file 1 — (DOCX 14 kb) [file 11121_2017_858_MOESM1_ESM.docx]

**Glossary**

**Average cost:** The total cost of an intervention divided by the number of participants.

**Benefit-cost analysis (BCA):** An economic evaluation approach where both the costs and benefits are monetized

**Benefit-cost ratio:** A ratio of the total intervention benefits divided by the total intervention costs

**Break-even analysis:** An analysis conducted as part of a benefit-cost analysis to determine the length of time until the benefits are equal to the costs.

**Budgetary impact analysis:** A form of cost-savings analysis that assesses the effects of an intervention on government spending or revenue for a specific agency, agencies or across the government.

**Cost:** The full economic value of the resources required to implement an intervention.

**Cost analysis (CA):** An economic evaluation approach that provides a comprehensive accounting of the economic costs of implementing an intervention

**Cost-effectiveness (CE) ratio**: A metric for summarizing the results of a cost-effectiveness analysis by dividing the intervention costs by the units of an outcome prevented by an intervention (e.g., lives saved, cases avoided)

**Cost-effectiveness analysis (CEA):** An approach for contextualizing the outcome of an intervention in terms of its cost. The cost per unit of outcome is calculated (see cost-effectiveness ratio). This ratio is compared to a standard measure of cost-effectiveness (e.g., another intervention’s cost-effectiveness ratio or a societal willingness to pay).

**Cost-savings analysis:** An economic evaluation where the impact of the program is only assessed from the government’s perspective. Specifically, the value of the intervention’s costs are compared to the value of the benefits to the government sector.

**Discount rate:** An adjustment rate used to estimate the value of costs and benefits incurred in the future at a equivalent value as costs and benefits in the present.

**In-kind cost:** Resources provided to support implementation that may originate from a separate source then the primary funding. Such costs are often donated goods or volunteered time and represent important resources that should be included in final cost estimates.

**Ingredients-Based Method**: A method for identifying, quantifying and valuing specific resources needed to deliver an intervention.

**Marginal cost:** The incremental cost to produce one additional unit of output (e.g., serve one more participant) given current costs..

**Market price:** The price that reflects an individual’s or society’s willingness to pay for an outcome of interest.

**Monetize:** The conversion of an intervention’s impact on an outcome from a nonmonetary metric to monetary terms.

**Monte Carlo simulation:** An approach for modeling uncertainty in an estimate that involves repeated sampling of of uncertain parameters from a study sample in order to create a distribution of possible outcomes. In economic evaluation this is often used for sensitivity analyses to create confidence intervals around estimates.

**Net present-value (NPV) benefits:** A summary metric that describes the present value of an intervention’s benefits minus the present value of the costs to deliver the intervention.

**Return on investment (ROI):** An economic evaluation where a benefit-cost analysis is conducted for a specific stakeholder’s perspective (e.g., private sector)

**Sensitivity analysis:** A post hoc analysis used to model or describe the uncertainty in an estimate.

**Shadow price:** The value of a resource as estimated when no market price is available or when market prices do not exists of capture the true cost

**Societal perspective:** A perspective taken in an economic evaluation that includes all other stakeholder perspectives.

**Start-up cost**: Resources consumed during the initial period of implementation that should be included in final cost estimates.

**Time horizon:** The span of time considered within an economic evaluation. This general includes the implementation period of a program until a set time-point of follow-up.

**Total costs:** The total sum of cost estimate by multiplying the quantity of all resources consumed by their corresponding unit costs.

Societal benefit
